# A Hardware-Friendly High-Precision CNN Pruning Method and Its FPGA Implementation

**DOI:** 10.3390/s23020824

**Published:** 2023-01-11

**Authors:** Xuefu Sui, Qunbo Lv, Liangjie Zhi, Baoyu Zhu, Yuanbo Yang, Yu Zhang, Zheng Tan

**Affiliations:** 1 Aerospace Information Research Institute, Chinese Academy of Sciences, No. 9 Dengzhuang South Road, Haidian District, Beijing 100094, China; 2 School of Optoelectronics, University of Chinese Academy of Sciences, No. 19(A) Yuquan Road, Shijingshan District, Beijing 100049, China; 3 Department of Key Laboratory of Computational Optical Imagine Technology, CAS, No. 9 Dengzhuang South Road, Haidian District, Beijing 100094, China

**Keywords:** convolutional neural networks, hardware friendly, network compression, regular pruning, LR tracking, high parallelism

## Abstract

To address the problems of large storage requirements, computational pressure, untimely data supply of off-chip memory, and low computational efficiency during hardware deployment due to the large number of convolutional neural network (CNN) parameters, we developed an innovative hardware-friendly CNN pruning method called KRP, which prunes the convolutional kernel on a row scale. A new retraining method based on LR tracking was used to obtain a CNN model with both a high pruning rate and accuracy. Furthermore, we designed a high-performance convolutional computation module on the FPGA platform to help deploy KRP pruning models. The results of comparative experiments on CNNs such as VGG and ResNet showed that KRP has higher accuracy than most pruning methods. At the same time, the KRP method, together with the GSNQ quantization method developed in our previous study, forms a high-precision hardware-friendly network compression framework that can achieve “lossless” CNN compression with a 27× reduction in network model storage. The results of the comparative experiments on the FPGA showed that the KRP pruning method not only requires much less storage space, but also helps to reduce the on-chip hardware resource consumption by more than half and effectively improves the parallelism of the model in FPGAs with a strong hardware-friendly feature. This study provides more ideas for the application of CNNs in the field of edge computing.

## 1. Introduction

In recent years, convolutional neural networks have become the most important intelligent algorithms in the field of computer vision [1,2,3,4], driving the implementation of artificial intelligence applications and being widely used in edge computing scenarios such as real-time target detection in cell phones, smart health devices, automobiles, drones, and satellites [5,6,7,8,9]. However, with the increasing detection accuracy requirements, the size of the network continuously expands with more and more parameters. For example, the number of parameters of the VGG-16 network [1] is about 130 million, the model storage is more than 500 Mb, and more than 30 billion calculations are required to complete one image detection task of 224 × 224 pixels. The large number of parameters leads to the need for off-chip memory, such as DDR, when deploying convolutional neural networks for smart chips with small on-chip storage space. The performance bottleneck of the off-chip memory is the data transfer delay, which can slow the data supply. During the operation of a CNN, frequent readings of the parameters in the memory are required, and the mismatch between the rates of data reading and calculation can cause the computational module to fail to achieve the expected efficiency and affect the system performance [10]. The huge amount of computation also leads to the challenge of deploying algorithms in smart chips with limited computational resources and I/O ports. Meeting the requirements regarding low power consumption, high accuracy, and high computational efficiency is difficult in edge applications.

**Figure 1:**
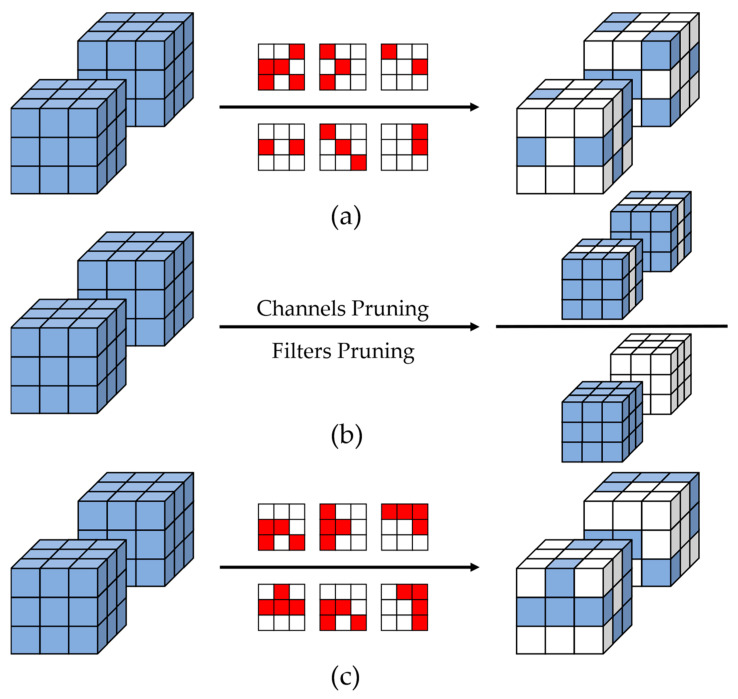
CNNs pruning methods: (**a**) non-structured pruning; (**b**) structured pruning; (**c**) pattern pruning. The blue cubes indicate the parts of the network parameters that are retained, and the white cubes indicate the part that is pruned away.

Therefore, improvements in CNN algorithms are essential to reduce the number of parameters and calculations. One approach is to directly design lightweight networks having fewer parameters and calculations, such as MobileNet [11], which uses depth-wise separable convolution instead of standard convolution and reduces the number of parameters by 33× and calculations by 27× compared with those of VGG-16. However, during the hardware deployment process, the computation of depth-wise separable convolution requires a large amount of memory access, which leads to problems such as high energy consumption and low efficiency. Another effective method is to directly prune the original CNN models [12,13,14,15,16,17,18,19,20]. By pruning the weights or structure in network models, the number of redundant parameters in the model can be reduced to reduce the amount of model computation and storage. During the hardware deployment process, using standard convolution in the conventional CNN model can considerably reduce memory access through mature data reuse technology [21,22]. At present, the mainstream CNN model pruning methods are usually divided into three types: non-structured [12,13,14,15], structured [16,17,18,19,20,23], and pattern [24,25,26] pruning, as shown in Figure 1.

The object of non-structured pruning is weights. The number of weights is sufficient to ensure a large amount of pruning without affecting the accuracy, and by randomly pruning the weights [12], we can obtain a high pruning rate model with high accuracy. Han et al. [12] first proposed the concept of nonstructured pruning to prune the AlexNet model by 80% with only a 0.1% accuracy loss. Liu et al. [14] proposed a weight regeneration retraining method, which resulted in the ResNet-50 pruning accuracy exceeding the baseline model with an 80% pruning rate, showing that nonstructured pruning achieves an excellent lightweight effect at the software level. However, for hardware deployment, the role of CNN model pruning can only be reflected by skipping all zero calculations [27]. The non-structured pruning method separates software from hardware and does not consider the continuous transmission and computation characteristics of data flow in hardware. The irregular weight distribution in the pruned model makes it difficult for hardware to directly skip the zero calculations, and the pruned model needs to be recoded and stored by compressed sparse rows (CSR), compressed sparse columns (CSC) [28], or some other special methods [27,29,30] to help the hardware select the input feature data that need to be computed according to the index to skip the zero calculation. Instead, these encoding methods bring a large amount of extra storage and large number of extra indexing operations, markedly reducing the computational efficiency and increasing the deployment difficulty. Moreover, random pruning leads to a different number of weights remaining in each convolutional kernel in the pruned model. In chips, such as FPGAs, with a highly parallel architecture [31], this feature can make the workload unbalanced between the computational modules of different channels, leading to the underuse of hardware resources and failure to take advantage of the lightweight network after pruning. Conversely, structured pruning is devoted to pruning with greater granularity, which is mainly divided into filter pruning [17,18] and channel pruning [19,20,23], which directly change the structure of CNN models by removing the filter and convolutional channels with higher regularity. This not only reduces the number of parameters and calculations, but also effectively reduces the generated intermediate feature map results, alleviates the pressure on data storage and transmission in the chip, facilitates hardware deployment, skips the zero calculations, and has good hardware adaptability. However, the structural changes caused by large granularity pruning have a large impact on the network, and the accuracy loss is difficult to recover, preventing high pruning accuracy at a high pruning rate. Li et al. [17] found that structured pruning can usually only maintain accuracy without descent at a pruning rate below 30% through a variety of experiments. Many subsequent researchers [18,19] performed structured pruning by different methods aiming to further increase the pruning rate, but they generally could only obtain a result of no decrease in accuracy at a 40% pruning rate. Ding et al. [20] were able to increase this number to 50% by introducing convolutional reparameterization methods, and their ResRep algorithm has become the SOTA of structured pruning. However, for a network with a huge number of parameters such as VGG, a pruning rate of 50% is still not enough.

To combine the advantages of both methods, pattern pruning was proposed [24,25,26]. Pattern pruning aims to find an intermediate sparse dimension to combine the high accuracy of small-grained pruning models with the high regularity of large-grained pruning models. The object of pattern pruning is also the weights, but it selects some specific convolutional kernel pruning patterns by analyzing the importance of each weight, and pruning is performed strictly according to these patterns. Tan et al. [26] achieved lossless pruning with a 60% pruning rate according to this approach. Actually, pattern pruning only reduces the number of convolutional kernels’ pruning patterns for nonstructured pruning and guarantees the same number of residual weights for each convolutional kernel, solving the problem of unbalanced workload between computational modules of different channels during hardware deployment. However, the weight distribution is still irregular, and the number of indexes needed for storage is only relatively reduced. To skip the zero calculations, additional indexing and input feature data selection operations are still required, which prevents the computational efficiency from being effectively improved. In other words, the current pruning algorithms cannot balance the software pruning performance and hardware deployment performance of CNNs, which has certain limitations.

Furthermore, to recover the accuracy loss from pruning, many researchers have used a classical pruning architecture with training, pruning, and retraining [12]. The conventional traditional retraining method usually chooses to fix the final learning rate during the original CNN training as the learning rate for the whole retraining phase [32]. During network training, a small learning rate leads to slow algorithm convergence and a large learning rate will make the algorithm scatter [33]. Smith [34] stated that a changing learning rate is the most beneficial for CNN training. To further increase the pruning accuracy and enhance the retraining effect, using a changing learning rate in the retraining process may be a solution.

To address the above issues, in this study, we focused on software and hardware co-optimization, considering a model pruning method with high sparsity, high accuracy, and high computational efficiency for easy deployment based on the different characteristics of the three pruning methods mentioned above. First, we developed a pruning method based on the row scale of convolutional kernels, KRP. The KRP method prunes each convolutional kernel according to the importance of each row, and each convolutional kernel retains only one row weight while all the remaining weights are pruned. The pruning granularity of KRP is between non-structured and structured pruning, and it is easy to obtain higher accuracy than that of structured pruning at the same pruning rate. Meanwhile, the KRP method has strong hardware adaptation capability. It ensures the same number of weights remain in each convolutional kernel, like pattern pruning, to avoid the problem of an unbalanced workload. The weight distribution of the convolutional kernels after row-scale pruning has high regularity, which can directly skip all the zero calculations produced by pruning by selecting the row from which the input feature data are entered during the hardware deployment process. Second, we propose a retraining method based on learning rate tracking to replace the conventional retraining approach in the retraining phase. This method reinitializes the learning rates in the retraining phase to their values in the learning rate schedule of the original CNN training process and corresponds the learning rates of the remaining epochs to the values in the schedule to achieve the effect of LR tracking, which can obtain higher training accuracy than conventional retraining in most cases. The whole process is similar to the retraining method proposed in the lottery hypothesis [35,36], where the unpruned weights are reinitialized to their original weights during the training process. Eventually, we performed a 4-bit quantization of the KRP pruned model using the GSNQ quantization proposed in our previous study [37] and designed a highly pipelined high-performance convolutional computation module on the FPGA platform for the obtained lightweight network to verify the hardware-friendliness of the KRP method. This module directly skips all the zero calculations without excessive indexing, significantly saves hardware resources, and improves computational efficiency. The results of our experiments with hardware and software verify that the proposed pruning algorithm can effectively achieve a balance between hardware and software performance, providing a hardware-friendly CNN pruning model with high accuracy.

In summary, this study provides a reference for the lightweight deployment of convolutional neural networks in edge applications from both theoretical and experimental aspects. The main work in this study was as follows:

1. In this study, we designed a CNN pruning method based on convolutional kernel row-scale pruning. It is highly practical by combining the high pruning rate and high accuracy features of non-structured pruning, high regularity and hardware- friendly features of structured pruning, and same number of remaining weights in each convolutional kernel of pattern pruning.
2. In this study, we developed a retraining method based on LR tracking, which sets the retraining learning rate according to the variation in the original training learning rate, which can more quickly achieve higher training accuracy than conventional retraining methods.
3. In this study, we performed pruning experiments on the CIFAR10 classification dataset [38] on four CNN models dedicated to CIFAR10, AlexNet [39], VGG-16 [1], ResNet-56, and ResNet-110 [40], and compared the results with those of state-of-the-art methods. We conducted comparison experiments on two commonly used training learning rate variations [1,40,41] to verify the effectiveness and generality of the proposed LR tracking retraining method.
4. In this study, we combined the KRP pruning method with our previously developed GSNQ quantization algorithm to propose a hardware-friendly high-precision CNN compression framework, which can match the original network performance while compressing the network by 27×. We designed a highly pipelined convolutional computation module on an FPGA platform based on this compression framework, which can skip all the zero calculations without excessive indexing and significantly reduce hardware resource consumption.

The remainder of this paper is organized as follows: Section 2 describes the proposed KRP pruning and LR tracking retraining methods. Section 3 describes the experimental details, and we analyze the experimental results. Section 4 provides a summary of the study.

## 2. Proposed Method

### 2.1. Convolutional Kernel Row-Scale Regular Pruning (KRP)

**Figure 2:**
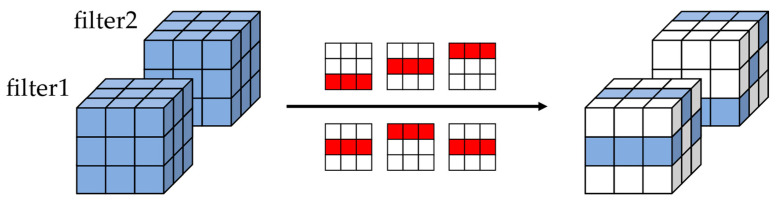
Convolutional kernel row-scale regular pruning (KRP).

Nonstructured pruning and pattern pruning cannot efficiently skip all the zero calculations during hardware deployment, and the random distribution of weights in the convolution kernel caused by unstructured pruning can lead to poor use of the hardware resources. In contrast, structured pruning with high regularity cannot achieve large-scale pruning and match the performance of the original network. To balance the advantages of these methods and obtain a hardware-friendly pruning CNN model with high sparsity, high accuracy, and high regularity, we developed a regular pruning method, KRP, based on the row scale of convolutional kernels, where only one row of weight is kept in each convolutional kernel and all other rows are pruned. The KRP method is shown in Figure 2, where the white indicates the pruned rows and the blue indicates the unpruned rows.

The KRP method ensures that each convolutional kernel is transformed into a highly regular sparse matrix with a regular arrangement of zero weights after pruning. In terms of hardware deployment, all the zero calculations produced by pruning are skipped by directly determining which row the input feature map data start to enter according to the weight distribution, which can be efficiently processed by the hardware architecture without too much additional data storage or data indexing. This reduces the amount of storage while reducing the amount of computation and improving the computational efficiency of the deployed CNN models with hardware-friendly features. In terms of software level, the pruning granularity of the KRP pruning method is between that of nonstructured pruning and structured pruning and can obtain higher pruning accuracy more easily than structured pruning.

A common method for determining the pruning criteria, i.e., to choose which row in the convolutional kernel should be pruned, is to judge the absolute value of the weights. In many previous studies, researchers have usually assumed that the larger the absolute value of the weight, the more substantial its effect on CNN models; and the smaller the absolute value of the weight, the less its effect on CNN models [12,42,43]. Based on this experience, many non-structured pruning methods use pruning criteria that directly remove weights with small absolute values [12,13,14]. Additionally, many researchers have explored the feasibility of the absolute value criteria in structured pruning methods [17,19]. A common evaluation criterion in structured pruning methods is to characterize the importance of a filter or kernel by calculating the sum of the absolute values of all weights in the whole filter or kernel (i.e., the L1 norm). A larger L1 norm indicates that this part has a stronger impact on CNN models. This evaluation criterion is the most effective model pruning criterion [13]. Therefore, the proposed convolutional kernel row-scale pruning method (KRP) is also characterized using this type of evaluation criterion.

Assuming that there is a total of *J* convolution kernels in a CNN model, for the *j*th convolution kernel $K_{j}$, with size *H* × *H*, we first cluster the weights in the convolution kernel by rows to obtain the *H* group of weight sets, and each group contains *H* weights. Then, we separately calculate the sum of the absolute values of all weights in each group of the weight set:

(1) $$S_{h}=\overset{H}{\underset{i=1}{\sum }}|W(i)|\;1\;\leq \;h\;\leq \;H$$

where $S_{h}$ denotes the sum of the absolute values of all weights in the *h*th group of weight sets in the kernel, and *W*(*i*) denotes the weights in the *h*th group of weight sets.

**Figure 3:**
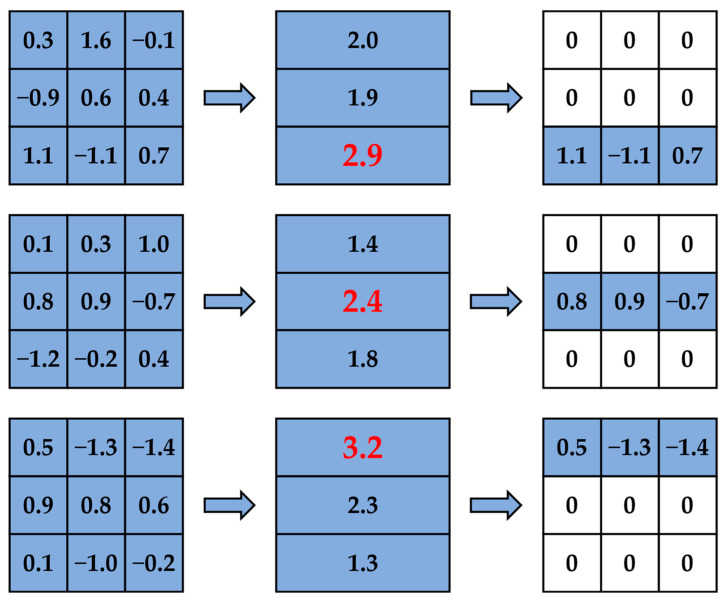
The KRP pruning criteria.

After calculating the absolute value of all the weight sets, we sort all the $S_{h}$ in each kernel, keep the row with the largest value of $S_{h}$, and set the weights in the other rows to zero to obtain the pruned convolutional kernel. The whole process is shown in Figure 3, where the red numbers indicate the largest values of $S_{h}$.

For CNN models that contain both convolutional and fully connected layers, the convolutional layers contain a large number of calculations, while the fully connected layer contains a large number of parameters. Taking the VGG-16 for the ImageNet dataset [44] as an example, the fully connected layers contain 89% of the parameters in the entire network model, while the convolutional layers contain only 11%. Conversely, the fully connected layers contain only 1% of the MAC operations of the entire network model, while the convolutional layer contains the remaining 99% [45]. This indicates that the convolutional layers are suitable for computational acceleration, and the fully connected layers have greater compression potential. Therefore, for all convolutional layers in CNN models, we pruned them using the proposed KRP regular pruning method to facilitate the acceleration of the CNN during hardware deployment. For all fully connected layers, we use the flexible and random unstructured pruning method based on the importance of weights [12], which has no excessive impact on computational efficiency and can obtain higher pruning accuracy.

### 2.2. LR Tracking Retraining

Many advanced CNN pruning methods follow a standard pruning framework: training the original CNN model; pruning the CNN model according to different pruning criteria; fine-tuning the pruning model by additional multiple retrainings with a low learning rate (LR) [32]. Because the accuracy of the CNN model is affected in the case of a large number of modifications or reductions in the original CNN parameters, it needs to be helped by means such as retraining to compensate for the accuracy loss. In this study, we also followed this pruning framework by fine-tuning the pruning model with retraining to restore network accuracy.

In the study of the lottery hypothesis [35], a high-performance subnetwork (which can be the pruning CNN model) was found during the CNN initialization phase. By initializing all the weights pruned off in the CNN models to their initial weights and then retraining, high-precision pruning results can be obtained at a faster speed. This study provided a new idea for the retraining of CNNs. Combined with the fact that a changing learning rate is more effective than a fixed one, as mentioned by Smith [34], we developed a retraining method based on LR tracking.

**Figure 4:**
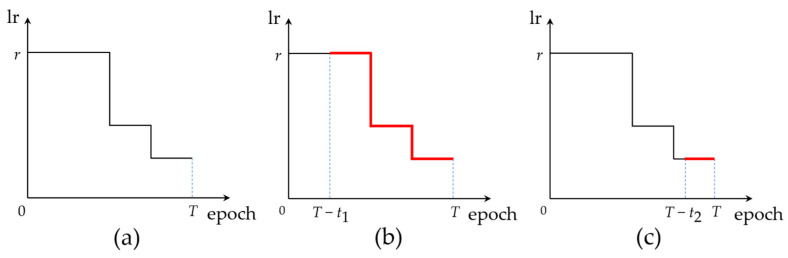
LR tracking retraining process: (**a**) learning rate schedule of original network training for T epochs; (**b**) learning rate schedule of LR tracking retraining for $t_{1}$ epochs; (**c**) learning rate schedule of LR tracking retraining for $t_{2}$ epochs.

We trained an original CNN model for T epochs to obtain a pre-trained model, recorded the learning rate schedule of this training process, and then pruned this model by the KRP pruning method proposed in Section 2.1 to obtain a new pruned CNN model. At this point, we set a retraining process of t epochs to recover the accuracy. We set the first learning rate of the retraining process as the learning rate at the T–t epoch in the original training learning rate schedule, and the subsequent retraining learning rate is directly set according to the change in learning rate after the T–t epoch in the original training learning rate schedule. This method is similar to tracking the learning rate of the original network training, which can be called LR tracking retraining and is shown in Figure 4.

The horizontal axis indicates the number of training epochs, the vertical axis indicates the corresponding learning rate, r is the initial learning rate of the original network training, and the black line indicates the preset learning rate variation pattern. T indicates the original network training epochs, $t_{1}$ and $t_{2}$ indicate two different settings for LR tracking retraining epochs and the red line indicates the learning rate variation pattern during LR tracking retraining. Figure 4b,c show that the LR tracking retraining is equivalent to tracking the learning rate schedule of the original network training after regressing the learning rate to the corresponding position.

A weight update phase occurs in the retraining process, and we need to restrict the pruned weights to keep the value of 0 in this process. Therefore, in this study, we set a mask matrix $M_{j\;}$ for each convolution kernel, defined as:

(2) $$M_{j}\;(a)=\{\begin{array}{cc} 0 & W_{j}\;(a)\;\in {\;P}_{j}\\ 1 & \mathrm{other}\;\mathrm{cases}\; \end{array}$$

where $W_{j}\;(a)$ denotes the weight *a* in the *j*th kernel, and $P_{j}$ denotes the set of pruned weights in the *j*th kernel. This formula is used to construct a structure identical to the original network by setting up a matrix containing only 0 s and 1 s, with 0 s denoting pruned weights, and 1 s denoting unpruned weights. When the network is retrained and the weights are updated, we use the stochastic gradient descent algorithm (SGD) and restrict the update of weights using the constructed mask matrix structure. The specific update method is as follows:

(3) $$W_{j}^{\prime }{\;(a)=W}_{j}\;(a)\;-\;\gamma \frac{\partial E}{\partial {(W}_{j}\;(a))}M_{j}\left(a\right)$$

where γ denotes the training learning rate under the corresponding epoch, *E* denotes the loss function, $W_{j}\;(a)$ denotes weight *a* in the *j*th kernel, $W_{j}^{\prime }\;(a)$ denotes the new weight after updating, and $M_{j}\left(a\right)$ denotes the mask corresponding to weight *a* in Equation (2). When $M_{j}\;(a)=1$ (i.e., the corresponding weights are the unpruned weights), the weights are updated normally during the retraining process; when $M_{j}\;(a)=0$ (i.e., the corresponding weights are the pruned weights), the weights are kept fixed during the retraining process without updating. In the accuracy recovery phase, the pruned weights are kept unchanged, and only the unpruned weights are retrained to compensate for the accuracy loss caused by pruning. This is the same for the fully connected layers.

For CNNs where all convolutional kernels are 3 × 3, such as VGG-16, ResNet-18, ResNet-56, ResNet-110, etc., the pruning rate of the KRP method is relatively low and proposed method easily compensates for accuracy loss. Therefore, for these CNN models, we used a one-time pruning method in this study to complete the pruning of the whole CNN at once, and then retrained based on LR tracking to recover the accuracy, which can significantly reduce the time cost of training. The whole KRP algorithm is shown in Algorithm 1.
**Algorithm 1** One-time KRP pruning process.**Input 1:** The pre-trained CNN model: ${\{W}_{j\;}:\;1\;\leq \;j\;\leq \;J\}$
**Input 2:** Learning rate schedule of the original CNN training process: ${\{\mathrm{LR}}_{t}\;:\;1\;\leq \;t\;\leq \;T\}$
1:  **for** j ∈ [1, …, J] **do**2:   Determine the L1 norm of all rows by Equation (1)3:   Keep the row with the largest L1 norm, prune the other weights, and update $M_{j}$
4:  **end for**5:  **if** there are FC layers6:   Set the pruning rate of FC layers for unstructured pruning and update $M_{j}$
7:  Set the LR tracking retraining epoch and retraining learning rate to correspond to ${\mathrm{LR}}_{t}$
8:  Update the weights with Equation (3)**Output:** Row-scale regular pruning CNN model

**Figure 5:**
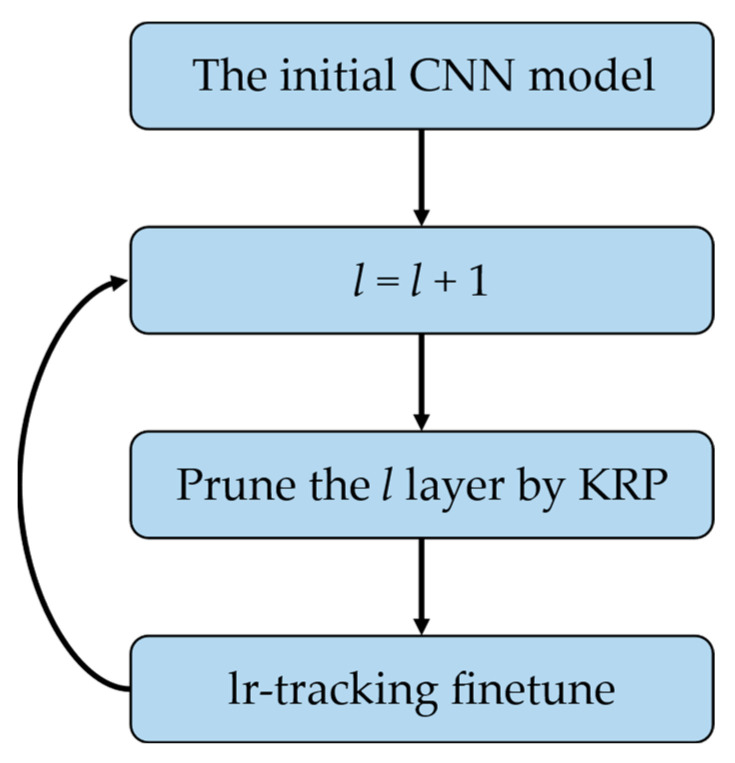
The incremental layer iteration pruning framework.

However, one-time pruning may not be suitable for CNNs containing a large number of 5 × 5, 7 × 7, or 11 × 11 convolutional kernels (GoogleNet [46], AlexNet [39], etc.). The accuracy loss with pruning by keeping only one row of weights in each kernel is too large to be eliminated. Therefore, an incremental layer iteration pruning framework needed to be introduced when necessary to complete pruning layer-by-layer, as shown in Figure 5.

### 2.3. Hardware-Friendly CNN Compression Framework Based on Pruning and Quantization

**Figure 6:**
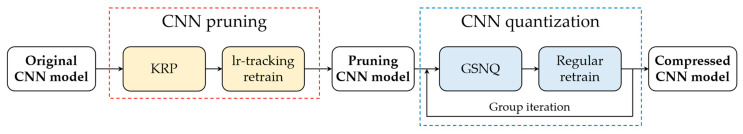
High-accuracy CNN compression framework.

In our previous study, we developed a hardware-friendly, high-accuracy, power-of-two quantization method, GSNQ [37], which can produce 3-bit or 4-bit high-accuracy quantization CNN models. Additionally, the power-of-two quantization achieves 0 on-chip DSP resource occupation in FPGAs, which effectively improves the CNN computational efficiency. We pruned the CNN model by the KRP method proposed in this study and then performed 4-bit quantization of the pruned model using GSNQ. Both methods are hardware-friendly, and the combination forms a hardware-friendly CNN model compression framework, which can achieve a compression rate of 26× to 27×. Figure 6 shows the overall framework of this CNN compression framework, where the pruning part uses a one-time pruning method, and the quantization part uses a grouped iterative method.

### 2.4. FPGA Design

**Figure 7:**
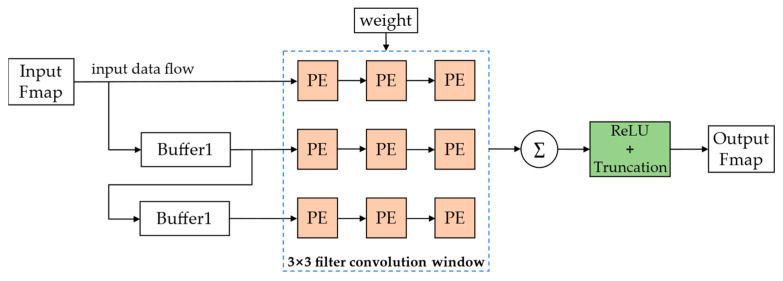
Convolutional computation module for CNN model based on GSNQ quantization.

A highly pipelined shift-operation-based convolutional computation module designed in our previous study [37] for the power-of-two quantization model is shown in Figure 7.

**Figure 8:**
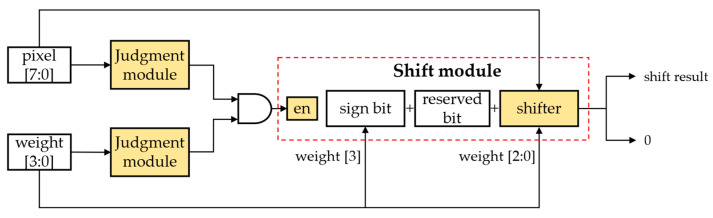
Multiplication processing unit based on shift operation.

This convolutional computation module functions to complete the convolution of an input feature map with a 3 × 3 convolutional kernel and outputs an output feature map after the ReLU activation function. PE is the multiplication processing unit based on the shift operation, as shown in Figure 8.

CNNs containing a large number of multiplication operations and the on-chip DSP resources in FPGAs being relatively scarce lead to a fundamental limitation of the computational efficiency of the CNNs in hardware. The designed multiplication processing unit uses shift operations instead of multiplication operations according to the characteristics of power-of-two quantization and can be directly implemented in FPGAs using abundant on-chip LUT resources for implementation, which can achieve the effect of not occupying any on-chip DSP resources and improving the computational efficiency.

**Figure 9:**
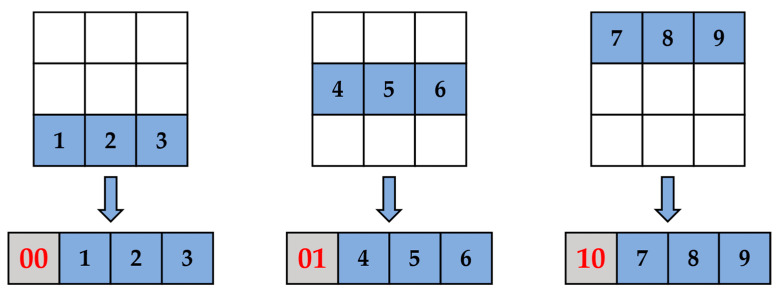
Rearranging weights.

For the CNNs compressed by the pruning and quantization-based network compression framework in this study, the structure in Figure 7 needs to be further improved by designing a special convolutional computation module in FPGA to adapt to the changed network structure after pruning. First, for the 3 × 3 convolutional kernels, there will be three weight distributions after KRP pruning. We need to rearrange and add indexes for them to efficiently calculate, as shown in Figure 9.

As shown in Figure 9, we convert the pruned 3 × 3 convolutional kernels to a 1 × 4 format for storage and computation in FPGAs. In addition to removing all the pruned weights, we also use 2-bit binary numbers (00, 01, and 10) as indexes to represent the three forms of pruned kernels, and we add them to the first position of the rearranged kernels. These indexes can facilitate the selection and computation of kernels in subsequent operations. Similarly, for pruned convolution kernels of other sizes, the corresponding $\left\lceil \;\log_{2}^{K}\right\rceil$-bit indexes are used to represent them, where *K* denotes the number of rows of convolution kernels, and ⌈ · ⌉ denotes the ceiling operation.

**Figure 10:**
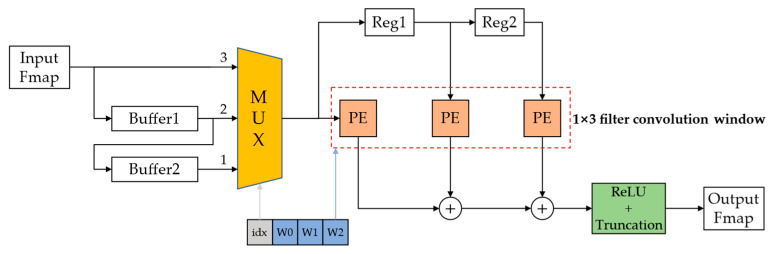
Convolutional computation module for CNN compression model based on KRP pruning and GSNQ quantization.

The rearranged convolution kernels are stored in the FPGA on-chip SRAM. Then, we designed a dedicated convolutional computation module to more accurately match the proposed KRP pruning method in a pipeline form according to the weight distribution characteristics of row-scale pruning, as shown in Figure 10.

Figure 10 shows that the input part is the same as that shown in Figure 7. We still use two buffers with a depth equal to the width of the input feature map to reuse the input feature map data (the number of buffers depends on the size of the convolutional kernel). After the module starts working, the feature map data start to be transferred into Buffer2 in the form of a data stream. After Buffer2 is filled, the remaining data start to be transferred into Buffer1, and when Buffer1 is filled, three input data streams are formed. After a three-to-one multiplexer (MUX), only one data stream is finally selected for subsequent calculations. Streams 1, 2, and 3 represent the data input from the first, second, and third rows of the feature map, respectively. For the three pruned convolutional kernel formats, when the index of the kernel in this convolutional computation module is 00, Figure 9 shows that the first two rows in the feature map will not be computed because the weights of the first two rows are all zero. This is equivalent to the 1×3 convolutional kernel starting calculating from the third row of the feature map, which represents the index 00 that corresponds to data stream 3. This means that after filtering by the MUX, we can skip all the zero calculations and only calculate the unpruned part. Similarly, the convolutional kernel with index 01 corresponds to data stream 2, and the convolutional kernel with index 10 corresponds to data stream 1. The first index of each rearranged kernel is used as the enable signal of the MUX to select its output data stream, which is input to the 1 × 3 convolution sliding window. PE is the multiplication computation unit based on the shift operation shown in Figure 8. We set two registers between the PEs to temporarily store the feature map data, and each register can output and input one feature data per clock cycle, which enables data reuse during convolution calculation and turns the 1 × 3 convolution kernel into a sliding window for sliding calculation in the feature map. The other ReLU activation functions and truncation module operations remain consistent with the design of our previously reported method [37].

This convolutional computation module takes advantage of the row-scale convolutional kernel pruning to significantly reduce the computation amount and storage requirement, while still maintaining a highly pipelined computation mode. Unlike unstructured pruning, this convolutional computation module can skip all the zero calculations produced by pruning without abundant extra indexes and retrieval operations, which can save more hardware resources and improve computational efficiency, and has strong practical application value.

## 3. Experiments

To verify the performance and universality of the row-scale convolutional kernel pruning and LR tracking retraining, we set up comparative pruning experiments to compare the proposed methods with a variety of other methods. In terms of hardware, to verify hardware adaptability, impact on CNN computation efficiency, and application value of the KRP method, we also set up several groups of comparative experiments on the designed convolution computation module on FPGAs.

### 3.1. KRP Pruning Comparative Experiments

#### 3.1.1. Implementation Details

To prove the effectiveness and universality of the proposed KRP method and LR tracking retraining at the software level, we set up pruning experiments on the CIFAR10 dataset on the AlexNet, VGG-16, ResNet-56, and ResNet-110 CNNs, which were all dedicated to CIFAR10, and compared the results with the traditional retraining method and existing representative pruning algorithms.

**Figure 11:**
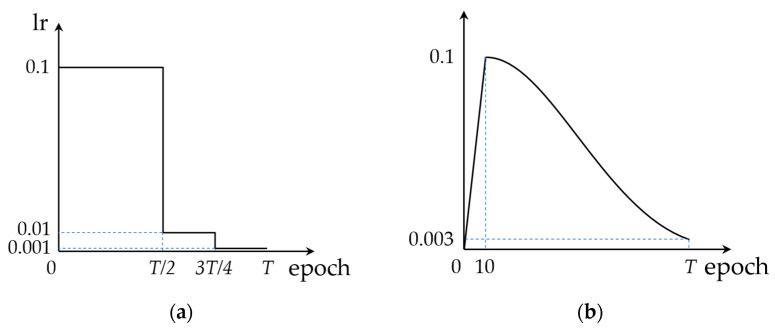
Learning rate change modes for original CNNs training: (**a**) standard step descent mode; (**b**) warm-up and cosine annealing attenuation mode.

We trained these original CNN models on the CIFAR10 dataset with two learning rate change modes and obtained the baseline model for retraining algorithm testing. One mode of learning rate was the standard training mode mentioned in the literature [1,40], and the other was a commonly used mode based on warm-up and cosine annealing attenuation [41]. The two learning rate change modes are shown in Figure 11.

For the two modes, we set the original training process for 160 and 110 epochs, respectively, for AlexNet and VGG-16; and 180 and 160 epochs, respectively, for ResNet-56 and ResNet-110. Other important training parameters are shown in Table 1.

The sizes of the convolutional kernels in VGG-16, ResNet-56, and ResNet-110 are all 3 × 3, while the sizes of the convolutional kernels in AlexNet include 11 × 11, 5 × 5, and 3 × 3. Therefore, for AlexNet, we adopted the incremental layer iteration framework shown in Figure 5, and we kept only one row of weights in all convolutional kernels with different sizes. For the other three networks, we used one-time pruning and retraining to reduce the time cost. We adopted a hybrid pruning method with KRP pruning for the convolutional layers and nonstructured pruning for the fully connected layers, so that the pruning rate of AlexNet and VGG-16 reached 70% and that of ResNet-56 and ResNet-110 reached 66.7%.

The whole experiment was based on the Python PyTorch library [47]. The development environment was the PyCharm Community Edition 2021.2.3, and the experimental platform was an NVIDIA GeForce RTX 2080 Ti GPU.

#### 3.1.2. Comparative Experiments of LR Tracking Retraining Method

**Figure 12:**
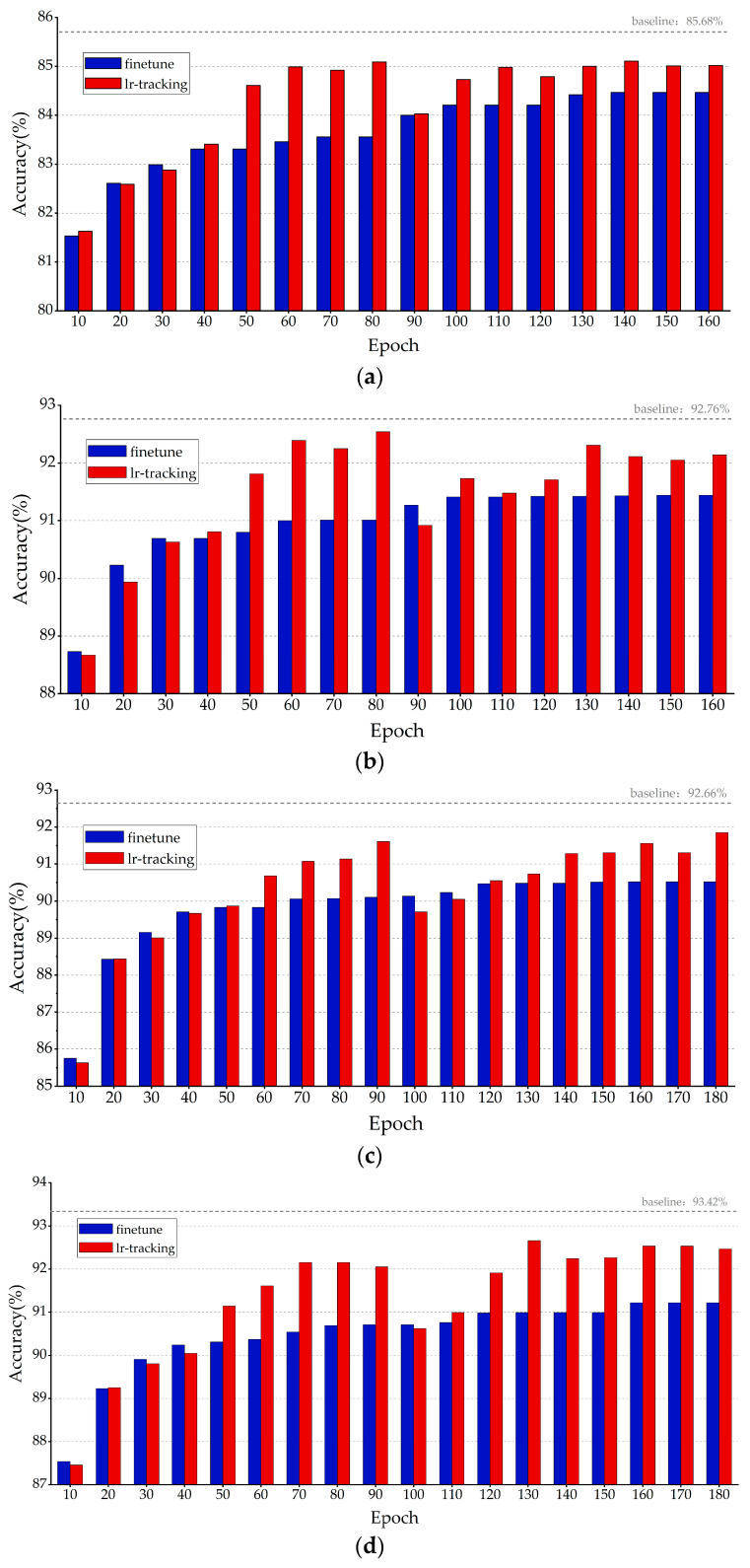
Results of different retraining methods with standard step descent training mode. Comparison of retraining results of (**a**) AlexNet KRP pruning model; (**b**) VGG-16 KRP pruning model; (**c**) ResNet-56 KRP pruning model; and (**d**) ResNet-110 KRP pruning models.

In this study, we separately conducted conventional traditional retraining and LR tracking retraining experiments for the obtained pruned models. For each CNN model, we set the retraining comparative experiments every 10 epochs between 0 and the total epochs of the original CNN training, and the results of the experimental comparison of the four networks under the standard step descent training mode are shown in Figure 12, where the blue indicates the highest retraining accuracy of the pruning model obtained by conventional retraining with different epoch numbers, and the red indicates the highest retraining accuracy of the pruning model obtained by the proposed LR tracking retraining with different epoch numbers. Each set of red and blue bar charts represents a set of comparison experiments.

**Figure 13:**
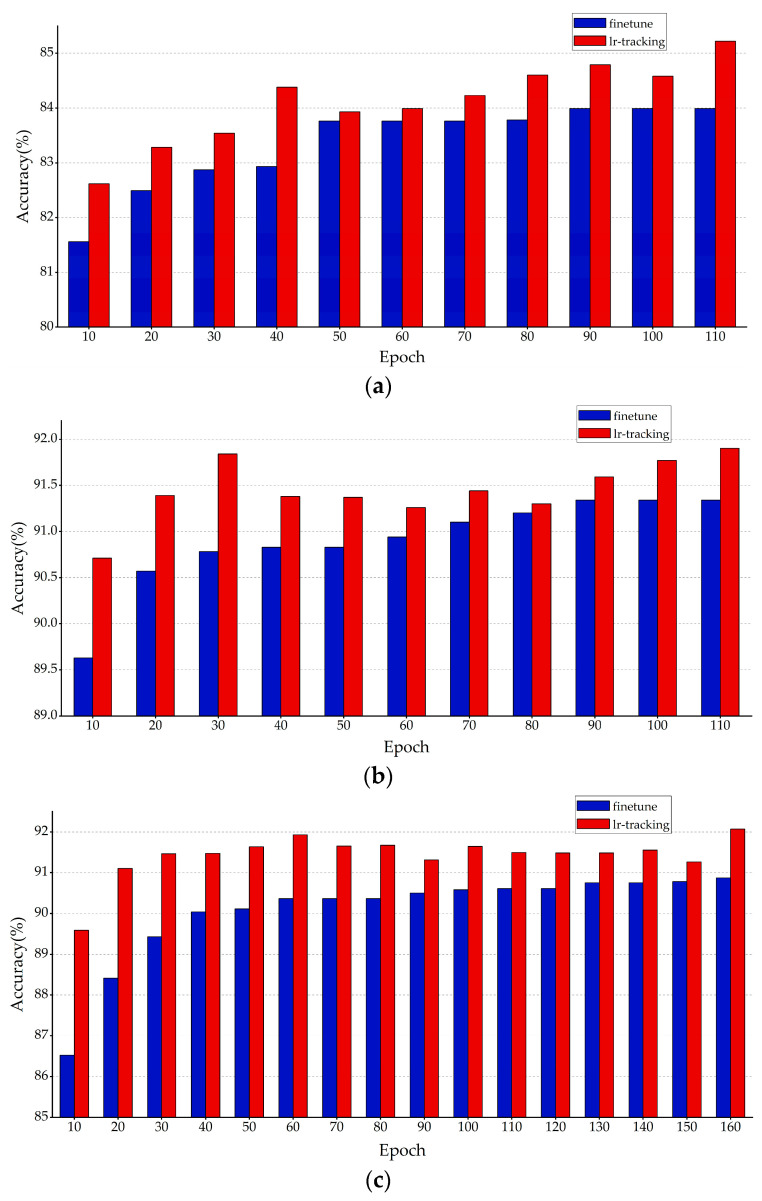

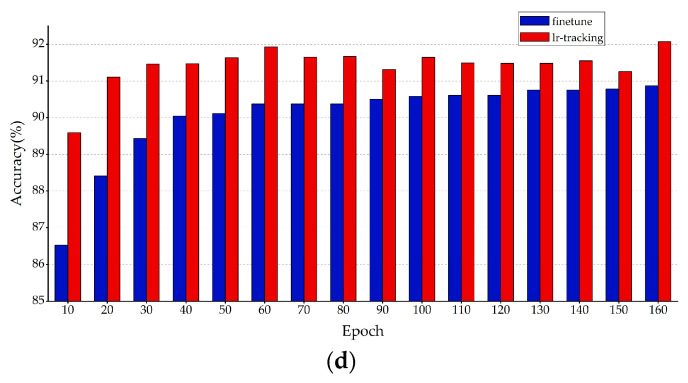
Results of different retraining methods with the warm-up and cosine annealing attenuation training mode. Comparison of retraining results of the (**a**) AlexNet KRP pruning model; (**b**) VGG-16 KRP pruning model; (**c**) ResNet-56 KRP pruning model; and (**d**) ResNet-110 KRP pruning model.

Figure 12b–d shows that for networks where the kernel sizes are all 3 × 3, one-time pruning was sufficient to obtain a high-performance pruning model at a pruning rate of 70% with an accuracy loss of no more than 0.8%. The results in Figure 12a show that for networks containing 11 × 11 and 5 × 5 convolutional kernels, such as AlexNet, the incremental layer iteration approach effectively compensated for the accuracy loss caused by large-scale pruning of large-size convolution kernels, and the final pruning accuracy loss was 0.58%. This provides strong proof that the proposed KRP pruning algorithm is applicable to any size of the convolutional kernel and has strong universality and effectiveness. The results in Figure 12 and Figure 13 show that the LR tracking retraining method outperformed the conventional retraining method in most cases, and the optimal settings of the epoch numbers for LR tracking retraining ranged from 35% to 50% and 70% to 100% of the total epochs of the original CNN training. The training performance at other epochs was similar to that of conventional retraining. From 0% to 35%, the learning rate variation in the LR tracking retraining method under the standard step descent training approach, as shown in Figure 11a, was the same as that of the conventional retraining method, resulting in close performance between them. The similarity in performance in the range of 50–70% illustrates that the network should not be trained at learning rates with fewer epochs followed by a direct reduction in the learning rate, which leads to the optimal value being skipped and affects the training accuracy.

Figure 13 shows a comparison of the results of the retraining experiments of the warm-up and cosine annealing attenuation training mode. Again, the blue indicates the highest retraining accuracy obtained by the conventional retraining, and the red indicates the highest retraining accuracy obtained by the LR tracking method. The results showed that the LR tracking retraining method outperformed the conventional retraining method at any retraining epoch in the original training mode of warm-up and cosine annealing attenuation.

**Figure 14:**
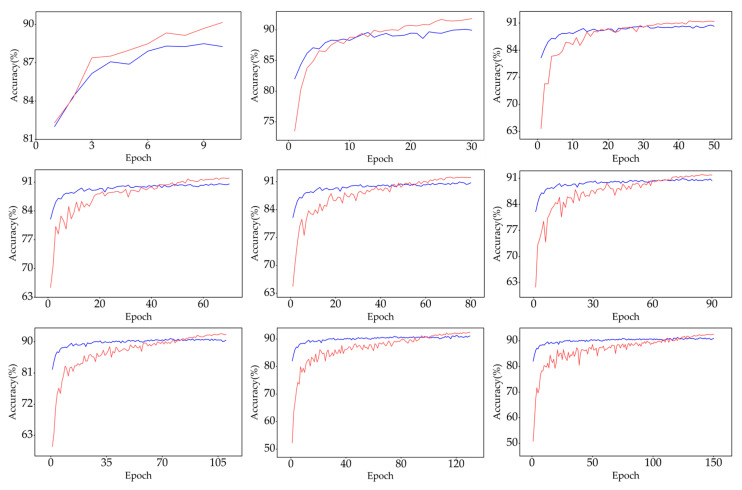
Specific training situation of two retraining methods.

Figure 14 shows the retraining accuracy variation in the results of the ResNet-56 KRP pruning model shown in Figure 13c for different epochs for the two retraining methods, with the blue solid line indicating the conventional retraining method and the red solid line indicating the LR tracking retraining method. The starting retraining accuracy was higher when using a fixed small learning rate to retrain, and it quickly converged and reached the optimal accuracy. The LR tracking method started with lower retraining accuracy, but the accuracy increased faster and finally always exceeded the accuracy of the conventional retraining method.

In conclusion, the LR tracking retraining method can obtain higher accuracy more quickly than the conventional retraining method, which can effectively reduce the time required for retraining and can be used as an alternative to the conventional retraining method. Additionally, with the LR tracking retraining method, the performance of the proposed KRP pruning algorithm is excellent in a variety of CNNs and can produce a hardware-friendly pruning CNN model with high accuracy and a high pruning rate, which has high practical value.

#### 3.1.3. Comparison with State-of-the-Art Pruning Methods

To further validate the proposed pruning method, we compared the results of KRP with those of some classical pruning methods [14,17,20,35,48] at similar pruning rates (all using one-time pruning), and we comprehensively evaluated them. Among them, the ResRep algorithm is the current SOTA model for structured pruning [20]. The comparison results are shown in Table 2.

The non-structured pruning algorithm ITOP had the highest pruning accuracy at the same compression rate. However, due to the considerable randomness of the weight distribution in the nonstructured pruning model, the obtained pruning model is not suitable for deployment in hardware and has low practical application value. The structured pruning model has strong practical application value because of its stronger pruning regularity, which does not require additional computation during, and is convenient for, hardware deployment. However, the experimental results showed that the pruning accuracy of the structured pruning algorithms (Lottery ticket, TRP, ResRep, etc.) was generally low. In contrast, the pruning network obtained by our KRP pruning method had high regularity, similar to that of structured pruning, while being more accurate than that of the structured pruning SOTA model ResRep. The accuracy loss of the final pruning model of KRP was less than 0.8%, essentially achieving one-time lossless pruning with a 70% pruning rate. We also verified in ResNet-56 that the KRP pruning method could exactly obtain a performance matching that of the baseline model at a 63.8% pruning rate, which is much higher than the 50% pruning rate of the structured pruning SOTA model [20].

In conclusion, the proposed pruning algorithm successfully solves the contradiction between the hardware and software performance of currently structured pruning and nonstructured pruning. On the premise of hardware-friendly, our algorithm has higher pruning accuracy at a high pruning rate, which demonstrates its strong application value.

#### 3.1.4. Experiments on CNN Compression Framework Based on KRP and GSNQ

We combined the KRP pruning method with the GSNQ quantization method proposed in our previous study [37] to develop a hardware-friendly and high-precision CNN compression framework, as shown in Figure 6. All parameters in the CNN pruning model obtained by KRP were still 32-bit floating-point numbers, which hinders the deployment of FPGAs. We used the GSNQ quantization method to quantize the KRP pruning models by 4 bits to obtain 4-bit fixed-point CNN models with 70% fewer parameters, which compressed the storage volume of the CNN models by 27×. For example, the original storage volume of the VGG-16 model dedicated to CIFAR10 was 114.4 Mb, and after compression by this compression framework and rearrangement of the pruning kernels shown in Figure 9, the final network model file stored in the FPGA was only 4.7 Mb, which could be directly stored in the on-chip SRAM on FPGAs without using off-chip memory. The compression effect is extremely remarkable. Table 3 shows the compression accuracy of the three CNN models after this compression framework.

We found that after being compressed by this compression framework, the accuracy of the VGG-16 model exceeded that of the baseline model, while ResNet-56 and ResNet-110 also basically experienced no accuracy loss. Therefore, the proposed pruning and quantization-based CNN compression framework can help to obtain 4-bit fixed-point lossless compression CNN models with a pruning ratio of 70%, which substantially reduces the bandwidth, storage, and computational pressure in FPGAs without losing accuracy. This is important for the practical application of convolutional neural networks.

### 3.2. FPGA Design Experiments

#### 3.2.1. Implementation Details

On the FPGA platform, we compared the convolution calculation module based on KRP pruning and GSNQ quantization with other FPGA deployment modules. The input used in the experiment was the 32 × 32 pixels feature maps in the CIFAR10 dataset. The whole FPGA deployment experiment was written in Verilog hardware description language based on a ZYNQ XC7Z035FFG676-2I chip, the on-chip hardware resources of which included 171,900 LUTs, 343,800 FFs, and 900 DSPs. The development environment was the official Xilinx compilation environment: Vivado Design Suite-HLx Editions 2019.2.

#### 3.2.2. Results Analysis

First, we compared the two designed convolutional computation modules (Figure 6 and Figure 9) with two conventional convolutional computation modules on FPGA: Module 1 [49], which used on-chip DSPs to implement multiplication operations traditionally, and Module 2 [50], which used on-chip LUT to implement multiplication operations by using the hardware synthesis function in the Vivado compilation environment. Modules 1, 2, and 3 were all convolutional computation modules for the non-pruned models, and module 4 added the KRP pruning method to module 3 as a convolutional computation module for the pruned model. Table 4 shows a comparison of the hardware resource consumption when the four convolutional computation modules ran independently.

We found that our designed module 3 achieved zero on-chip DSP occupation compared with module 1, while the consumption of other resources remained basically the same. Compared with module 2, the on-chip LUT and FF consumption in module 3 was significantly reduced. These were described in detail in our previous study [37].

The proposed pruning and quantization compression framework performed better in terms of on-chip resource consumption. From the resource consumption of module 4, we can see that the KRP pruning method not only reduced the CNN parameters by a large amount, but also helped to reduce on-chip hardware resource consumption while maintaining efficient hardware operation. Compared with the convolution calculation module 3, which only quantized and did not prune, the hardware resource consumption of module 4 was reduced by more than 50%. Compared with modules 1 and 2, module 4 even significantly reduced hardware resource consumption overall, which means that the KRP pruning method can theoretically help to achieve more than twice the parallelism of the original CNN model and has additional advantages in multichannel parallel CNN deployment computation.

In most cases, the number of input and output channels of convolutional layers in CNNs was between 32 and 512. Therefore, in the FPGA deployment work, regardless of the method followed to accelerate the CNN computation, a convolutional computation accelerator architecture must be built with at least 32 convolutional computation modules in parallel [51,52,53,54,55]. Therefore, we also performed 32-channel parallel processing for the designed modules and compared the results of the four methods to verify the performance of the proposed method in real applications. Table 5 shows the on-chip hardware resource consumption of different modules for 32-channel parallelism.

We found that, as in the comparison of one convolutional computation module, the GSNQ quantization method well overcame the limitation of on-chip DSP resources on the deployment performance, and module 3 performed better than modules 1 and 2. The KRP pruning-based module 4 further significantly reduced the on-chip hardware resource consumption of FPGA deployment on this basis, occupying the fewest resources. Without the on-chip DSPs limitation, we could continue to stack 32-channel parallelism up to 64-channel or even 128-channel parallelism. The proposed KRP pruning method and designed convolutional computation module can considerably save the on-chip hardware resources of FPGAs and achieve increased parallelism to enable higher computational efficiency, which strongly demonstrates the hardware-friendliness and engineering application value of the proposed KRP pruning method. Although the two cases of quantizing parameters into 4-bit and 3-bit have less impact on the on-chip hardware resource consumption in FPGAs, considering the slightly larger accuracy loss of 3-bit quantization, the 4-bit quantization of the KRP pruning model using the GSNQ method in the proposed CNN compression framework is a comprehensive choice with outstanding performance.

## 4. Conclusions

In this study, we designed an innovative CNN regular pruning method, KRP, based on the row scale of convolutional kernels and a hardware-friendly and high-accuracy CNN compression framework. The KRP pruning method uses the rows in the convolutional kernel as the basic pruning unit, keeping only one row for each convolutional kernel and pruning all other rows. The pruning granularity is between that of nonstructured pruning and structured pruning, and it can easily obtain higher pruning accuracy than structured pruning methods with high regularity, and all the zero calculations caused by pruning can be skipped during FPGA deployment without much indexing. During model retraining, we proposed a special retraining method based on LR tracking, which can achieve far better results than traditional retraining methods by strictly tracking the learning rate schedule of the original CNN training. The results of several sets of experiments demonstrated that the KRP pruning method can produce lossless network pruning models at a pruning rate of over 60%. The proposed CNN compression framework based on the KRP pruning method and GSNQ quantization method can help to construct a lightweight CNN model that exactly matches the performance of the original model. For the compression models, we designed a high-performance convolutional computation module in FPGA. The results of multiple sets of experiments demonstrated that the KRP pruning method can help to significantly reduce the storage and hardware resource consumption during deployment in FPGA, and more effectively improve the computational parallelism and memory access efficiency with good hardware adaptation capability. The methods described in this paper provide a complete idea for the application of CNNs in the edge computing field, which can help with constructing high-accuracy lightweight CNN models and implementing a high-performance hardware accelerator.

## Figures and Tables

**Table 1:** Important CNN training parameters.

Network	Batch Size	Weight Decay	Momentum
AlexNet	64	0.0001	0.9
VGG-16	64	0.0001	0.9
ResNet-56	128	0.0001	0.9
ResNet-110	128	0.0001	0.9

**Table 2:** Performance comparison of different pruning methods on the CIFAR10 dataset.

Networks	Methods	Pruning Type	Pruning Rate	Accuracy	Δ Accuracy
VGG-16 (Baseline: 92.76%)	Pruning filters	Structured	69.7%	90.63%	−2.13%
	Lottery ticket	Structured	69.7%	92.03%	−0.73%
	ITOP	Nonstructured	70.0%	92.99%	+0.23%
	**KRP**	**Between both types**	**70.0%**	**92.54%**	**−0.22%**
ResNet-56 (Baseline: 92.66%)	Pruning filters	Structured	70.0%	90.66%	−2.00%
	Lottery ticket	Structured	70.0%	91.20%	−1.46%
	TRP	Structured	66.1%	91.72%	−0.94%
	ResRep	Structured	66.1%	91.84%	−0.82%
	ITOP	Nonstructured	66.7%	93.24%	+0.58%
	**KRP**	**Between both types**	**66.7%**	**91.87%**	**−0.79%**
	**KRP**	**Between both types**	**63.8%**	**92.65%**	**−0.01%**

**Table 3:** Accuracy by the CNN compression framework.

Network	Accuracy		
	Baseline	KRP	KRP and GSNQ
VGG-16	92.76%	92.54% (−0.22%)	92.79% (+0.03%)
ResNet-56	92.66%	91.87% (−0.79%)	92.56% (−0.10%)
ResNet-110	93.42%	92.66% (−0.76%)	93.28% (−0.14%)

**Table 4:** On-chip resource consumption comparison of one convolution computing module. Bit width indicates the bit width of the CNN parameters after GSNQ quantization.

Modules	Bit Width	LUTs	FFs	DSPs
Module 1: implement multiplication by on-chip DSPs	4 bits	375	293	9
Module 2: implement multiplication by on-chip LUTs	4 bits	499	257	0
**Module 3: based on GSNQ only**	**4 bits**	**397**	**273**	**0**
**Module 4: based on KRP and GSNQ**	**4 bits**	**162**	**165**	**0**
Module 1: implement multiplication by on-chip DSPs	3 bits	250	268	9
Module 2: implement multiplication by on-chip LUTs	3 bits	402	226	0
**Module 3: based on GSNQ only**	**3 bits**	**263**	**237**	**0**
**Module 4: based on KRP and GSNQ**	**3 bits**	**124**	**134**	**0**

**Table 5:** On-chip resource consumption comparison of 32-channel convolutional computing modules in parallel.

Modules	Bit Width	LUTs	FFs	DSPs
Module 1: implement multiplication by on-chip DSPs	4 bits	13,438 (7.82%)	11,463 (3.33%)	288 (32%)
Module 2: implement multiplication by on-chip LUTs	4 bits	16,906 (9.83%)	9960 (2.90%)	0 (0.00%)
**Module 3: based on GSNQ only**	**4 bits**	**13,917 (8.10%)**	**10,824 (3.15%)**	**0 (0.00%)**
**Module 4: based on KRP and GSNQ**	**4 bits**	**5481 (3.19%)**	**6051 (1.76%)**	**0 (0.00%)**
Module 1: implement multiplication by on-chip DSPs	3 bits	9430 (5.49%)	9938 (2.89%)	288 (32%)
Module 2: implement multiplication by on-chip LUTs	3 bits	13,849 (8.06%)	9037 (2.63%)	0 (0.00%)
**Module 3: based on GSNQ only**	**3 bits**	**9500 (5.53%)**	**9348 (2.72%)**	**0 (0.00%)**
**Module 4: based on KRP and GSNQ**	**3 bits**	**4231 (2.46%)**	**4899 (1.42%)**	0 (0.00%)

## Data Availability

Not applicable.
